# Development of African swine fever epidemic among wild boar in Estonia - two different areas in the epidemiological focus

**DOI:** 10.1038/s41598-017-12952-w

**Published:** 2017-10-02

**Authors:** Imbi Nurmoja, Katja Schulz, Christoph Staubach, Carola Sauter-Louis, Klaus Depner, Franz J. Conraths, Arvo Viltrop

**Affiliations:** 1 Estonian Veterinary and Food Laboratory (VFL), Kreutzwaldi 30, 51006 Tartu, Estonia; 2 Estonian University of Life Science, Institute of Veterinary Medicine and Animal Sciences, Kreutzwaldi 62, 51014 Tartu, Estonia; 3 Friedrich-Loeffler-Institut, Federal Research Institute for Animal Health, Institute of Epidemiology, Südufer 10, 17493 Greifswald-Insel Riems, Germany

## Abstract

African swine fever (ASF) in wild boar emerged in Estonia for the first time in September 2014. The first affected region was located in the South of Estonia close to the border with Latvia. It was considered to be epidemiologically connected to the outbreaks in the North of Latvia. About two weeks later, cases were detected in the North of Estonia, close to the Russian border. In the present study, we aimed to investigate the epidemiological courses of the disease in the South and in the North of Estonia. Potential associations between risk factors and the laboratory test results for ASF were examined. A hierarchical Bayesian space–time model was used to analyze the temporal trend of the ASF seroprevalence in the two areas. Young wild boar were statistically significant more likely to be ASF-positive by both, serology and virus detection, than older animals. A statistically significant difference between the two areas in the temporal course of the seroprevalence was found. While the seroprevalence clearly increased in the South, it remained relatively constant in the North. These findings led to the hypothesis that ASF might have been introduced earlier into the North of Estonia then into the South of the country.

## Introduction

African swine fever (ASF) is a notifiable viral pig disease whose emergence usually entails huge economic consequences for the pig industry^1^. In Europe, the disease affects both domestic pigs and European wild boar (*Sus scrofa*). Therefore, an infected wild boar population holds the constant risk to infect domestic pigs and vice versa^2^.

Apart from Sardinia, where ASF has been endemic since 1978, Europe was officially free from ASF since 1995^1^. However, ASF was newly introduced into Georgia in 2007. From there the virus spread to neighboring countries such as Armenia, Azerbaijan, the Russian Federation, Ukraine and Belarus.

The spread of the ASF virus p72 genotype II in eastern Europe has involved both domestic pigs and wild boar^3^. In 2011, the virus entered the central part of the Russian Federation, where it is now endemic^3,4^. In addition, several outbreaks in domestic pig were confirmed in Northwest Russia in the region of St. Petersburg between 2009 and 2012, about 160 km away from the Estonian border^4^.

In January 2014, the first ASF wild boar case was reported from Lithuania^5^. Subsequently, in the course of the year, Poland as well as Latvia confirmed ASF cases in wild boar^6,7^. Finally, Estonia officially reported the first ASF case in wild boar in September 2014.

**Figure 1:**
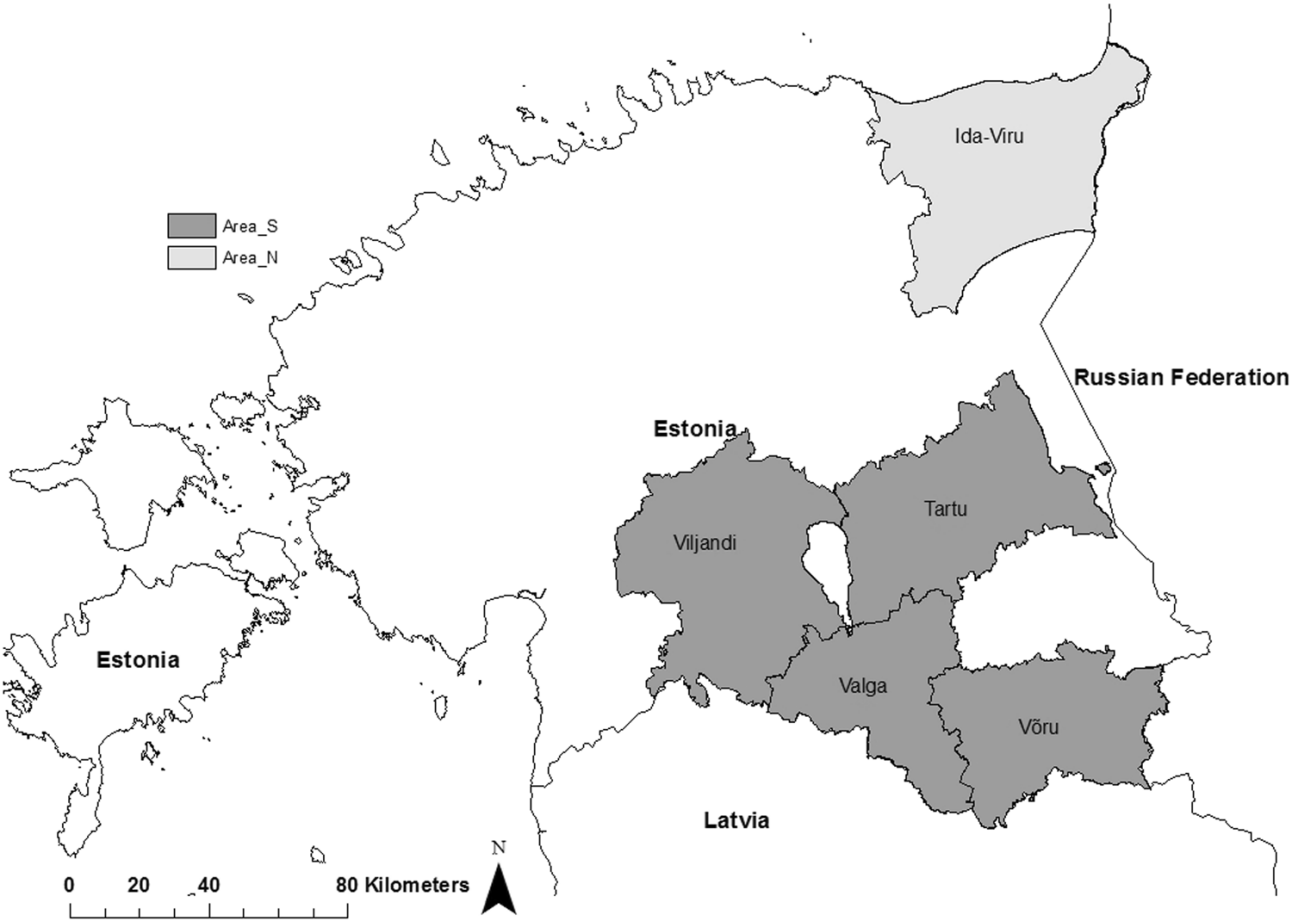
The study areas and the bordering countries in the South and East. Highlighted areas illustrate the four included counties in the South (area S) and the one in the Northeast of Estonia (area N). Map was generated by using ArcGIS ArcMap 10.3.1 (ESRI, Redlands, CA, USA, http://www.esri.com/).

The first ASF-positive dead wild boar in Estonia was reported on 2^nd^ September 2014 in Valga county, six km from the Latvian border^8^ (Fig. 1). One week later, the virus was detected in wild boar in Viljandi county, which is also bordering Latvia. The outbreaks in the South were most likely epidemiologically connected with the epidemic in the North of Latvia, which had started few weeks before^7^. On 14^th^ September 2014, an ASF-positive wild boar was found in Ida-Viru county, located in the Northeast of Estonia next to the border with the Russian Federation and more than 200 km away from the affected areas in the South^9^. The third county bordering Latvia, Võru county, was found infected by the end of October 2014.

By the end of 2014, 73 infected wild boar had been detected in Estonia; 69 of them in the southern region and four in the Northeast. In the first half of 2015, the disease largely remained in the infected areas. However, in the mid of 2015, it spread to previously uninfected areas. A total of 1,530 ASF cases in wild boar have been officially reported in Estonia until the end of September 2016^10^.

There was evidence suggesting that the course of the epidemic differed between the areas in the South and in the Northeast of Estonia. In the Northeast, the proportion of hunted animals that were virologically negative but seropositive was relatively high and almost no findings of dead wild boar were reported, while in the South a high mortality among wild boar was observed. In addition, in the South hunted animals found infected with ASF were mainly virologically positive, but seronegative, while in the North also seropositive wild boar were found^11^ (Table 1). Moreover, the spread of the disease in the South appeared to be more rapid as compared to the North, where the infection seemed to remain within one area. We found no obvious factors that may have caused differences in the reporting of fallen or hunted wild boar in these two regions. Hunting practices are similar and the ASF surveillance system as well as the reporting regulations are the same everywhere in Estonia.

**Table 1:** ASFV genome positive and -negative wild boar samples, averaged prevalences and 95% confidence intervals (calculated using R) for the study areas (N = study area North, N1 = first 12 months of the study period, N2 = second 13 months of the study period; S = study area South, S1 = first 12 months of the study period, S2 = second 13 months of the study period).

Area	Number of samples	Number of negative samples	Number of positive samples	Averaged prevalence within the study period (%)	95% CI
N	1,174	1,152	22	2.0	1.1–3.0
N1	353	351	2	0.8	0.2–3.5
N2	821	801	20	2.4	1.5–3.7
S	5,841	5,039	802	13.7	12.8–14.6
S1	2,670	2,301	369	13.8	12.5–15.2
S2	3,171	2,738	433	13.7	12.5–14.9

In the present study, we aimed to analyze available data and therefore improve our understanding of the epidemiology of ASF and the course of the epidemic in Estonia. We tested potential associations between risk factors such as age, population density and carcass category (i.e. wild boar found dead or hunted) and positive virological or serological laboratory test results as the outcome variable. However, our main aim was to evaluate the apparent epidemiological differences between the infected areas in the North and the South of Estonia. To ensure the comparability of these two areas, we tested the hypothesis that there was a difference in the age of wild boar or in the carcass distribution between the different study areas.

## Material and Methods

### Study area

Estonia is administratively divided into 15 counties (first level administrative division). The local governance is on municipality level (second level administrative division). Each county comprises of several municipalities (cities or towns and rural municipalities). During the study period 183 rural municipalities existed in Estonia.

We defined two different study areas in Estonia based on county level. The southern region (area S) comprised four counties (50 municipalities), namely Valga (2,044 km²), Viljandi (3,422 km²), Võru (2,305 km²) and Tartu (2,993 km²), of which the latter is the only one not bordering Latvia. The infected region in the Northeast (area N) bordering the Russian Federation included only one county (21 municipalities), Ida-Viru (3,364 km^2^) (Fig. 1).

### Sampling and sample analysis

Wild boar were sampled based on the Estonian animal disease control program and included both wild boar found dead and hunted animals. Wild boar found dead, including animals killed in road traffic accidents or shot sick, were sampled in the whole country irrespectively of the ASF status of the area (passive surveillance). However, the sampling scheme of hunted wild boar (active surveillance) changed several times depending on the ASF status of the affected area. These changes were due to updates of European Commission Implementing Decision 2014/709/EU. In practice, in areas where wild boar were affected by ASF (Decision 2014/709/EU, Part II), all hunted wild boar were sampled, whereas in areas at risk of getting infected, but without previous detection of ASF cases (Decision 2014/709/EU, Part I), approx. 2% of hunted wild boar were tested.

From hunted wild boar, blood samples were collected for ASFV genome and antibody detection by hunters immediately after hunting, whereas organ (kidney, spleen, lymph node) or bone marrow samples from animals found dead were collected for virus genome analysis by official veterinarians shortly after detection of the animals had been reported (within 24 hours). Although the quality of samples varied among all sample types, this had no significant impact on the performance of the PCR test. The test result was only reported as valid if correct test performance was confirmed, also by using an appropriate internal control. A total of 30 bone marrow and 57 serum samples were found unfit for PCR testing and therefore excluded.

Real-time PCR (used for virus genome detection), enzyme-linked immunosorbent assay (ELISA) and the indirect immunoperoxydase technique (IPT) (both used for antibody detection) were conducted at the Estonian Veterinary and Food Laboratory the National Reference Laboratory for ASF in Estonia. Real-time PCR was performed according to the protocol published by Tignon, *et al*.^12^. Although specific values for the diagnostic sensitivity and specificity of this protocol have not been published, a high sensitivity and a specificity of almost 100% can be assumed after extensive validation of the method^12,13^. A commercially available blocking ELISA (Ingezim PPA COMPAC, Ingenasa, Madrid, Spain) was used according to the manufacturer’s instructions (sensitivity: 98%, specificity: 100%). In the case of an inconclusive ELISA result, the sample was re-tested in the IPT for confirmation. If samples were tested by both ELISA and IPT, the outcome of the IPT was considered as the final result.

For IPT, a protocol provided by the European Union Reference Laboratory for ASF (CISA-INIA, Valdeolmos, Spain) with a sensitivity of 98.2% and specificity from 99.0% to 100% (when used as an individual test), was used. If samples were sent to the European Union Reference Laboratory, this test was also used for the detection of antibodies in organ and bone marrow samples^14,15^.

### Data

For the analyses, surveillance data from 1^st^ September 2014 until the 30^th^ September 2016 (25 months) were used. In addition, the study period was divided into two parts for the prevalence analyses in each study area (N and S). The virus prevalences and seroprevalences were not only analyzed for entire duration of the study period (25 months), but also separately for the first 12 and the last 13 months. Surveillance data of 2015 and 2016 were extracted from the CSF / ASF wild boar surveillance database of the EU Reference Laboratory (https://public.surv-wildboar.eu/Default.aspx). The data for 2014 were obtained from the database of the Estonian Veterinary and Food Laboratory. It comprised 1,957 data records in total. In the final set, data from counties outside the study area were removed. The data set finally used included information on the place (county and municipality level), year and month of sampling, age (assessed by the hunters) and the origin of wild boar (carcass: hunted or found dead), the virological and serological test results and the population density.

We used wild boar population data provided by the Estonian Environment Agency (Nature department). The data were collected using different methods, such as hunting bag statistics, snow-track counts and hunter estimation^16–18^. Population data were available of the hunting years 2013, 2014 and 2015. The numbers of wild boar were recorded at the end of the according hunting year in the pre-reproductive time (observation dates: march 2014, 2015 and 2016). Data were available as integer numbers per hunting district. A hunting district is defined as an area for big game hunt with a size of at least 5,000 hectares according to the Estonian Hunting Act^19^. To use the data for analyses, we aggregated them at the municipality level. Utilizing the software ArcGIS ArcMap 10.3.1 (ESRI, Redlands, CA, USA, http://www.esri.com/), the wild boar density per km² was calculated based on the estimated number of wild boar per hunting ground. The area of hunting grounds that overlapped with the territories of at least two municipalities, were proportionally attributed to the territory of each municipality. By means of the wild boar density per km² and the adapted hunting grounds, the total number of wild boar per municipality was calculated. Finally, wild boar densities were determined for each municipality.

### Statistical analyses

All statistical analyses were performed using the software package R (http://www.r-project.org)^20^. We estimated stratified period prevalences over time and space and calculated confidence intervals and odds ratios according to Clopper and Pearson^21^. A p-value of ≤ 0.05 was considered statistically significant.

To test for statistically significant associations between presumed risk factors and a positive virological or serological test results for ASF on the animal level, the Fisher’s exact test was performed using the whole data set. Accordingly, the potential association between age and the laboratory test results was investigated. The animals were attributed to the age classes “juvenile” (<1 year) and “adult” (>1 year). Potential associations between the carcass categories (“hunted” or “found dead”) and the laboratory test results were also examined. Furthermore, the age distribution within the two carcass categories was analyzed.

When testing for potential associations between the population density and positive ASF laboratory test results, the municipalities as the variable of interest were categorized depending on their test results (0 = only negative test results within the study period, 1 = at least one positive test result within the study period). Since the distribution of the data was not known, the non-parametric Mann-Whitney U test was used for statistical analysis. For this purpose, population densities were averaged over the reported years and assigned to each municipality. Due to lack of knowledge on the distribution of the data, the hypothesis that the population densities differed between the two study areas was also tested using the non-parametric Mann-Whitney U test.

The hypothesis that the age or carcass distribution was different between the study areas was examined using Fisher’s exact test. This test was also used to examine potential associations between the study areas and the virological or serological status of wild boar.

### Model analyses

To test for a temporal and spatial effect within the two study areas, a hierarchical Bayesian space–time model was used^22,23^. The model was only applied for the seroprevalence. The period for detecting the viral genome in hunted animals is generally short, which is likely to lead to false-negative results, i.e. animals that were ASF-positive, but not at the time of sampling or not in the available sample, have to be regarded as uninfected. Therefore, a stable trend analysis can only be performed with the serological results. The implementation of the model was adapted from the one described by Staubach, *et al*.^22^. Variables identified as statistically significant by univariable analyses were included as fixed effects, whereas space and time were treated as random effects. The analyses were conducted separately for each of the study areas (area N and area S) on municipality level using BayesX 2.0.1 (http://www.uni-goettingen.de/de/bayesx/550513.html). A Markov Chain Monte Carlo algorithm (MCMC) was applied to estimate the parameters of the model. Figures were generated by using the software package R (http://www.r-project.org)^20^ and maps created using the software ArcGIS ArcMap 10.3.1 (ESRI, Redlands, CA, USA, http://www.esri.com/).

## Results

### Data

After removing data from other counties then the study area, 7,015 data records were available for analyses. Within the study period of 25 months, 7,015 samples had been investigated virologically (Table 1) and 6,306 samples also serologically by ELISA. Only 319 samples were tested by IPT because the method had not yet been implemented in the beginning of the epidemic (Table 2).

**Table 2:** ASF antibody-positive and -negative wild boar samples, averaged prevalences and 95% confidence intervals (calculated using R) for the study areas (N = study area North, N1 = first half of the study period (12 months), N2 = second half of the study period (13 months); S = study area South, S1 = first 12 months of the study period, S2 = second 13 months of the study period).

Area	Number of samples	Number of negative samples	Number of positive samples	Averaged prevalence within the study period (%)	95% CI (%)
N	1,142	1,098	44	3.9	2.8–5.1
N1	338	313	25	7.4	4.8–10.7
N2	804	785	19	2.4	1.4–3.7
S	5,164	4,977	187	3.6	3.1–4.2
S1	2,315	2,281	34	1.5	1.0–2.0
S2	2,849	2,696	153	5.4	4.6–6.3

### Statistical analyses

A statistically significant association between age and the positive laboratory test results was found for both, real-time PCR and serology by ELISA/IPT (p < 0.001). Based on the results, the probability to detect an ASFV- or antibody-positive animal was higher in young animals ( < 1 year) (real-time PCR: OR = 1.57, 95% CI = 1.35–1.83; serology: OR = 1.89, 95% CI = 1.45–2.47). Also, regarding the carcass category (hunted or found dead), a statistically significant association was found (p-value < 0.001). The probability to find a real-time PCR- or antibody-positive animal was higher in animals found dead (real-time PCR: OR = 69.60, 95% CI = 56.89–85.15; serology: OR = 4.53, 95% CI = 2.83–7.25). No statistically significant difference in the distribution of the two age classes within the carcass categories was detected (p-value = 0.420). In both, hunted wild boar and those found dead, the proportion of old animals was slightly higher (see Supplementary Figure S1).

**Figure 2:**
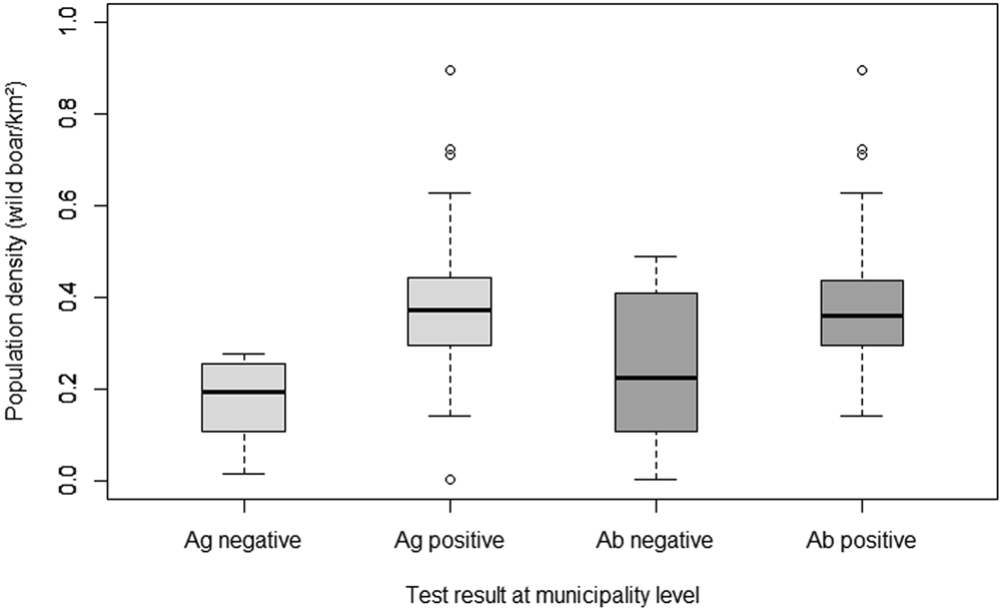
Population density (number of wild boar/km²) in the municipalities of the study areas stratified by the virological and serological test result at the municipality level. Ag: ASFV genome detection, Ab: antibody detection. Figure was generated by using the software package R (http://www.r-project.org)^20^.

A significant association was found between the wild boar population density and the test results regarding both ASFV genome detection by real-time PCR and serology (real-time PCR, p < 0.001; serology, p = 0.009). ASFV-positive municipalities had a higher population density than ASFV-negative ones (Fig. 2).

The age distribution of sampled wild boar was similar in areas S and N (p-value = 0.566) (see Supplementary Figure S2). However, the distribution of wild boar found dead and hunted animals was different (p-value < 0.001); in area S, the proportion of animals found dead was significantly higher than in area N (see Supplementary Figure S3). In area S, the population density was significantly higher than in area N (p-value < 0.001) (see Supplementary Figure S4).

The prevalence of ASFV genome-positive wild boar was significantly higher in study area S as comped to area N (p-value < 0.001). However, there was no significant difference in the seroprevalence between these areas (p-value = 0.728).

### Model analyses

Due to the results of the univariable analyses, namely the significant association between age, carcass category, population density and the serological test results, these factors were included in the hierarchical Bayesian space–time model as fixed effects. In area N, age and population density showed a significant effect on the serological test result, whereas in area S, age and carcass category, but not population density resulted in a significant effect on the test results (Table 3 and Table 4).

**Table 3:** Parameter estimates obtained from the Bayesian model for three factors in area North (N); BCI: Bayesian credible intervals.

Model	Mean	SD	Median (95% BCI)	Mean/St.Dev.*
Constant	−2.735	0.938	−2.687 (−4.678; −0.842)	
Carcass	−0.732	1.292	−0.620 (−3.708; 1.424)	0.567
Age	0.737	0.348	0.741 (0.062; 1.394)	2.122
Population density	−5.713	2.899	−5.573 (−11.841; −0.274)	1.971

DIC:323.82; Deviance:291.558; pD:16.135 *Mean/Std.Dev. >1.96, indicating statistical significance.

**Table 4:** Parameter estimates obtained from the Bayesian model for three factors in area South (S); BCI: Bayesian credible intervals.

Model	Mean	SD	Median (95% BCI)	Mean/St.Dev.*
Constant	−4.370	0.344	−4.371 (−5.081; −3.737)	
Carcass	1.533	0.342	1.544 (0.820; 2.100)	4.480
Age	0.580	0.173	0.579 (0.244; 0.924)	3.357
Population density	0.443	0.604	0.446 (−0.734; 1.600)	0.733

DIC:1344.465; Deviance:1269.215; pD:37.625 *Mean/Std.Dev. > 1.96, indicating statistical significance.

**Figure 3:**
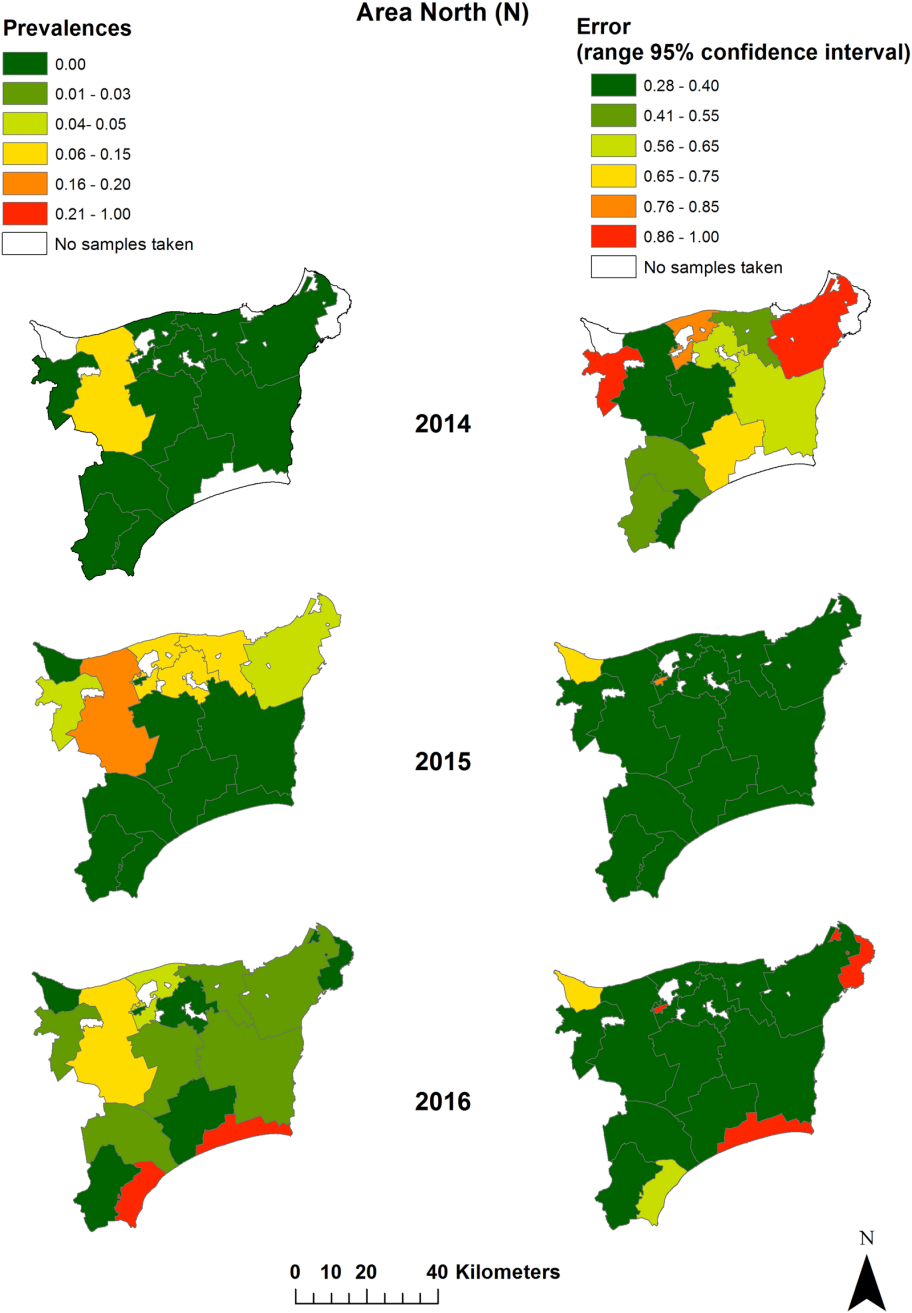
Seroprevalences and 95% confidence intervals for sampled wild boar per municipality in study area N (Ida-Viru county) in 2014 (Sept. – Dec.), 2015 (Jan. – Dec.) and 2016 (Jan. – Sept.). Maps were generated by using ArcGIS ArcMap 10.3.1 (ESRI, Redlands, CA, USA, http://www.esri.com/).

**Figure 4:**
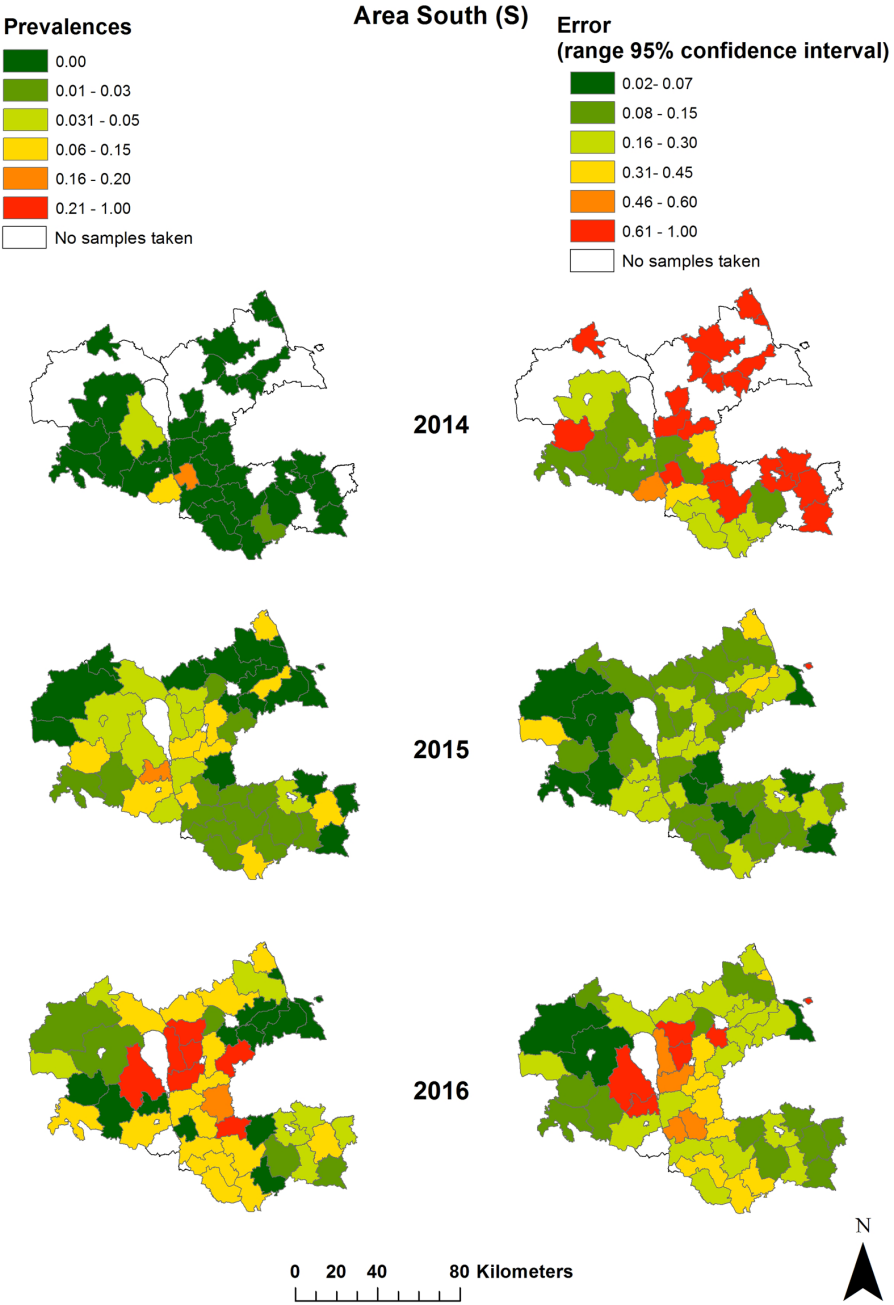
Seroprevalences and 95% confidence intervals for sampled wild boar per municipality in study area S (Viljandi, Tartu, Valga and Voru county) 2014 (Sept. – Dec.), 2015 (Jan. – Dec.) and 2016 (Jan. – Sept.). Maps were generated by using ArcGIS ArcMap 10.3.1 (ESRI, Redlands, CA, USA, http://www.esri.com/).

The analyses of sample sizes resulting from active surveillance at municipality level showed in both study areas that the sample sizes differed considerably among municipalities and over time (Figs 3 and 4). Spatial analysis on the basis of the Bayesian model confirmed a different trend of the seroprevalences within the two study areas, which was already evident from the raw prevalence data. In area N, the highest prevalences were observed in one municipality in the western part of Ida-Viru county over the entire study period. In 2015 (data of all 12 months were included in the analyses), the prevalences were also higher in municipalities located more east, but in 2014 (data of four months were included) the sample sizes were too small to obtain reliable prevalence estimates for these municipalities. In 2016 (data of nine months were included), the infection expanded also to municipalities located in the South of area N (Fig. 3). In area S, the infection spread over time within the wild boar population. In contrast to area N, the prevalences were high in the municipalities bordering Latvia in 2014 and in the course of the following years, an expansion of the affected areas towards the North occurred (Fig. 4).

In both areas, N and S, the small sample sizes have to be considered when interpreting the results.

**Figure 5:**
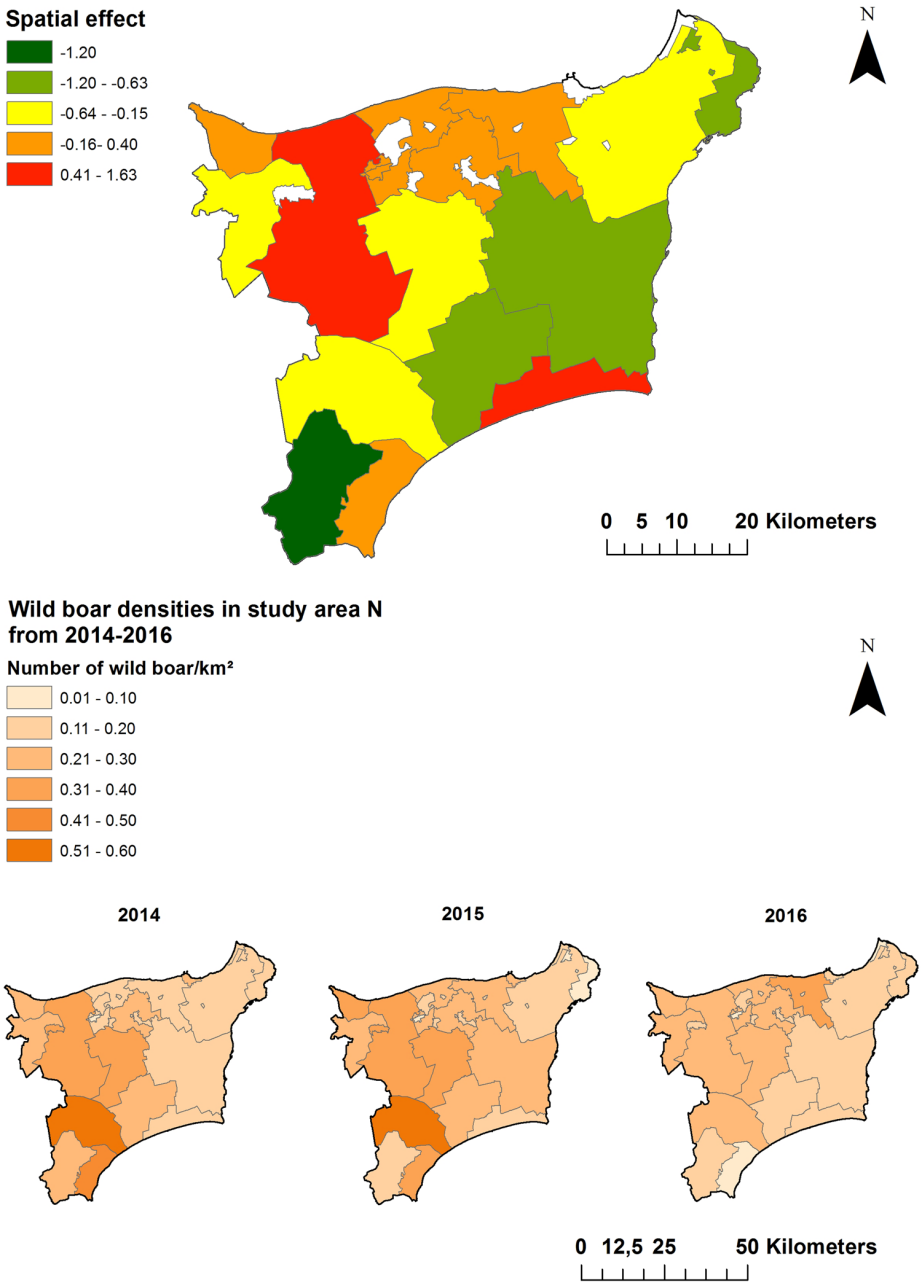
Median-structured spatial effect on the logit prevalence per municipality in study area N (Ida-Viru county) for the study period of 25 months. Maps in the lower row show the population density (number of wild boar/km²) for each municipality in study area N. Maps were generated by using ArcGIS ArcMap 10.3.1 (ESRI, Redlands, CA, USA, http://www.esri.com/).

The spatial analyses yielded a clear median spatial effect on the logit prevalence per municipality in the North of area N. In the eastern and very southern part of the county, a negative spatial effect was found. The wild boar population density was higher in the western part of area N as compared to the eastern area bordering Russia (Fig. 5).

**Figure 6:**
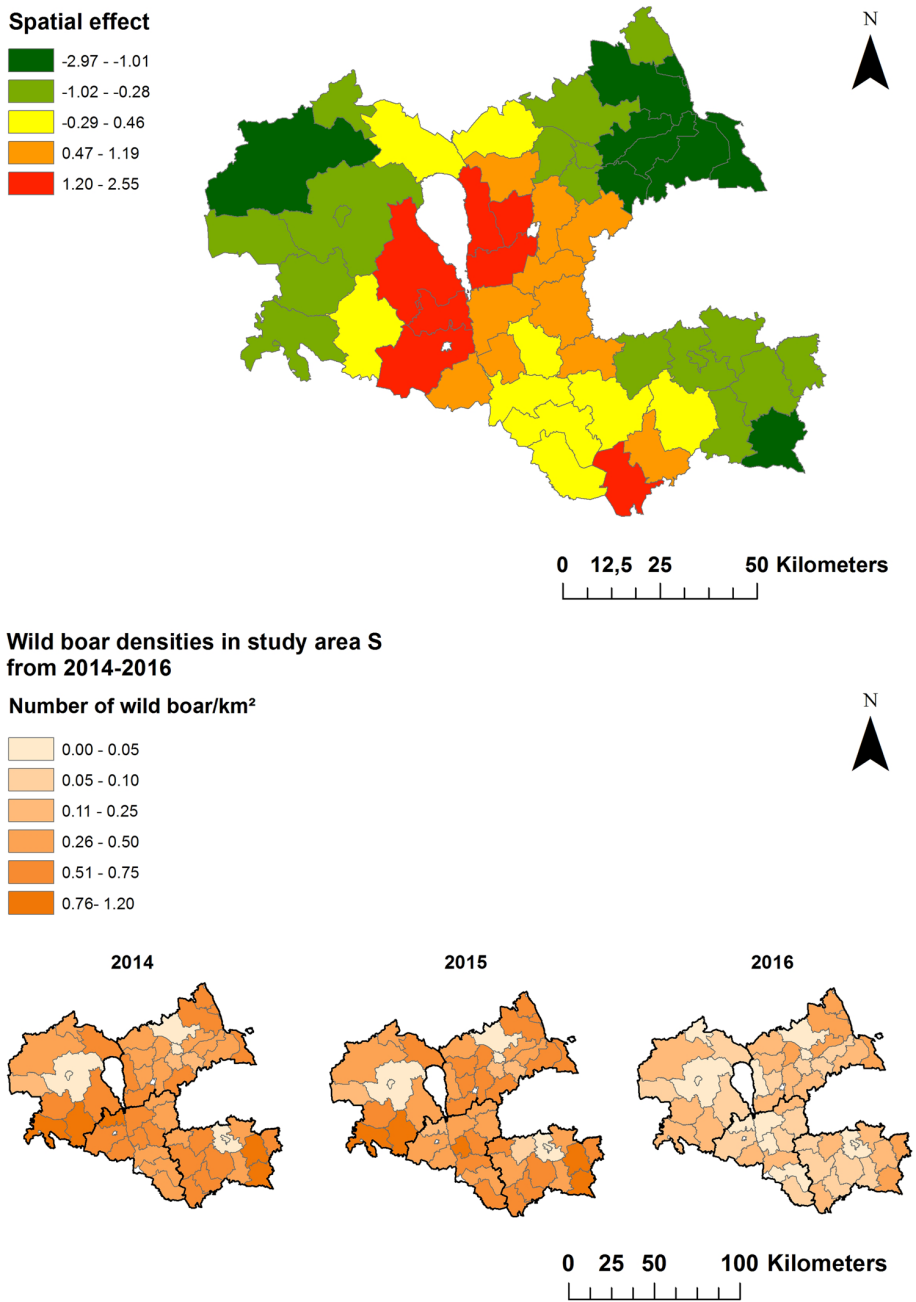
Median-structured spatial effect on the logit prevalence per municipality in study area S (Viljandi, Tartu, Valga and Voru county) for the study period of 25 months. Maps in the lower row show the population density (number of wild boar/km²) for each municipality in area S. Maps were generated by using ArcGIS ArcMap 10.3.1 (ESRI, Redlands, CA, USA, http://www.esri.com/).

In area S, the strongest dynamic of infection, shown by a structured spatial effect (Fig. 6), became evident in some of the municipalities bordering Latvia and the ones located further north. Negative spatial effects were seen in the municipalities in the West and the East of the study area (Fig. 6). In area S, the average population density was higher than in area N. In both areas, the population density decreased over time (Figs 5 and 6).

**Figure 7:**
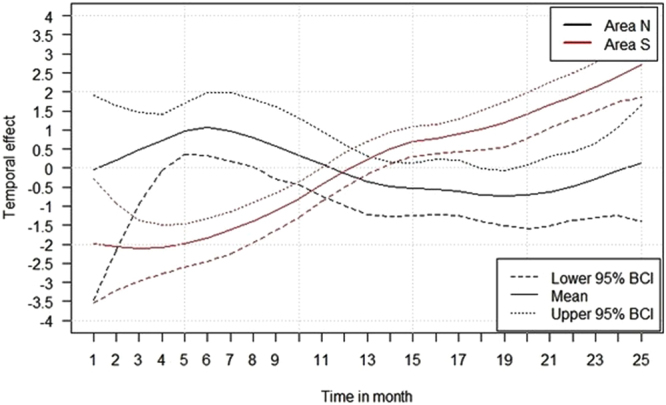
Median temporal effect on the logit prevalence in area North (N) and in area South (S) for the study period of 25 months. 95% Bayesian credible intervals (BCI) are included. Figure was generated by using the software package R (http://www.r-project.org)^20^.

The temporal analyses resulted in a significant difference of the median temporal effect on the logit prevalence between the two study areas. In contrast to area N, where no temporal effect was observed, a significant increasing trend during the whole study period of 25 months was seen in area S (Fig. 7).

## Discussion

When ASF emerged in Estonia in 2014, two different areas, namely in the North and in the South, were affected. Although the events in the South were connected with ASF outbreaks in the North of Latvia^7^, only Estonian data were analyzed. Variations in the course of the ASF epidemic in the two areas led to the hypothesis that the events might be independent and differ in their epidemiology. The aim of this study was to test this hypothesis and to describe the epidemiology of the ASF epidemic in wild boar in defined areas of Estonia.

The study area in the South comprised four counties with a total area of 10,764 km², whereas the study area in the North consisted only of one county with a size of 3,364 km². In the South, not only the area under investigation was bigger but also in that area the wild boar density was higher. Therefore, the number of investigated samples was higher in the South. Confidence intervals therefore need to be considered when interpreting the results. Furthermore, the observed incidence per spatial unit and time step is not a useful estimate of the underlying disease prevalence due to different sample sizes as well as temporal spatial dependencies between neighboring areas. By applying a hierarchical Bayesian space–time model, the extra-sample variation and spatial/temporal correlations in the data were accounted for. The chosen model is suitable to analyze data with gaps and particularly variable sample sizes per spatial and temporal unit^22,23^. To estimate the fitness of the model the deviance information criterion (DIC) was used^24^.

It was found that the probability to detect an ASFV genome- or antibody-positive animal was higher in young wild boar. This stands in contrast to the results of experimental studies, where no age-dependent degree of susceptibility could be detected^25,26^. However, recent experiments with a small number of animals showed that young animals survived long enough to develop antibodies, even in the case of acute-lethal courses of ASF. All these animals were also tested PCR positive^27^. Further field and experimental studies are therefore needed for clarification. Statistical analyses resulted also in a higher probability to find virologically and serologically positive animals in wild boar found dead than in hunted wild boar. This is very likely to be due to the high lethality of ASF. These findings once more emphasize the need of an increased effort to support passive surveillance and to encourage hunters to focus on the detection and sampling of dead wild boar^28,29^.

The present study demonstrated a statistically significant positive association between population density and the municipality status regarding ASF (by ASFV genome detection or serology). This may be due to the fact that in densely populated regions the transmission rate between wild boar is higher, since it is known that direct contact between wild boar is strongly beneficial for transmission of ASF^30–32^.

The findings regarding the association between age, carcass and population density and the serological test results were supported by analyses of virological data and the appropriate result, which showed the same associations. (IPT: specificity approximately 100%)^5^. Only 22 samples originating from 22 animals found dead showed a serologically positive test result, because laboratory routine procedures did not include antibody detection from organ and bone marrow samples. However, the strong association between animals found dead and a positive virological test result still point at the importance of detecting and sampling wild boar found dead^29^.

To be able to include the factor population density in the analyses, data had to be transferred from the hunting district level to the municipality level. The applied method certainly led to a slight deviation from true wild boar densities. However, the density data at the hunting district level are mere estimates of hunters, based on their account of the hunting bag. In addition, the population density is subject to constant change. The reliability of these data is therefore always a challenge. The available hunting data originated from the pre-reproductive period before most females give birth. Accordingly, it can be assumed that at another time point of data capture, the number of wild boar per km² would be clearly higher.

It was not surprising that the age distribution was the same in the area N and S. This result demonstrates that the population structure was similar in the two areas, which may be due to similar hunting practices. This justifies comparing the results of the laboratory investigations for N and S. The proportion of the sampled animals found dead was significantly higher in area S. The significantly higher average ASFV genome prevalence in area S may be seen as a result of the significantly higher number of animals found dead in study area S and their higher chance to be positive by ASFV genome detection.

The Bayesian model was only applied for serology. Due to the fact that ASFV in wild boar samples is only detectable over a very limited period of time^32^ and that no measurable memory effect is available, a trend analyses was not feasible with regard to the results of ASFV genome detection.

The results of the univariable analyses differed slightly from the ones obtained by Bayesian modelling. For the univariable analyses, this may be explained by the inclusion of the whole data set, independently of the study area whereas for the Bayesian model the data were analyzed for area N and area S separately. Also, data were adjusted for space and time in the model. Still, in both areas, the significant association between age and the serological result could be confirmed. In contrast to the univariable analyses, in area S, a significant association was shown between carcass category and serology. This might be due to the higher relative number of animals found dead in area S and accordingly their greater importance in the epidemics. Population density showed a significant effect on the seroprevalence in area N, which is consistent with the results of the univariable analyses. In area S, population density had no significant effect, which may be explained by the bigger size of study area S as compared to N and the associated heterogeneity of the population densities in the single municipalities.

The spatial effect on the logit prevalence indicates a difference between the respective courses of infection in the two study areas. In area N, the infection seemed to be stable in one area. In contrast, in area S, in 2014 the prevalences were high in the areas bordering Latvia and the infection seemed to move North over time. This spread may have been supported by the higher population density in area S, which makes a higher transmission rate likely^30^. Although the prevalence seemed to increase in the center of study area S, the width of the 95% CI was also increasing. This is probably due to the ASF-related decrease of the wild boar population in these municipalities over time and thus to the lower number of investigated samples. The findings of the spatial analysis also support the hypothesis that the infection was already present in area N for a longer period of time, whereas it was still spreading in area S at the time when the study was conducted. Accordingly, since the epidemic in the South did not reach its climax and did not stop spreading, it is impossible to prove these hypotheses at the moment. However, it would be advisable to re-analyze the situation in the two areas in one or two years again. The incidence of ASF currently seems to level off and no increase of seroprevalence is observed anymore, we expect that the situation in area S will then result in a similar picture as now observed in area N.

Although the average seroprevalence over the study period of 25 months did not differ significantly between the two areas, the temporal trend analysis showed a significant difference in the course of infection. The number of data sets per municipality and per analyzed time point was relatively small, but our data suggest that the trend varied between the two areas, also when on the Bayesian credibility intervals were taken into account.

The increase of the temporal logit prevalence in area S led to the assumption that ASF was newly introduced into that area, that naïve animals got infected and that the proportion of animals developing antibodies subsequently grew. By contrast, no temporal effect was seen in area N. These assumptions were supported by the results of the descriptive analyses. In study area S, the average seroprevalence showed an increase over time, whereas in area N the average prevalence of antibody-positive wild boar was even lower in the last 13 months of the study period. We therefore hypothesize that ASF may have been present a longer time period in area N before the start of the study period, i.e. before the first case was officially confirmed. This hypothesis is supported by the fact that several outbreaks had occurred in the St. Petersburg area^4^, located only 160 km away from the Estonian border and connected with Ida-Viru county through a highly frequented highway between 2009 and 2012. Furthermore, the very small sample sizes at the beginning of the study period (September 2014) and the ones of 2012, 2013 and of the beginning of 2014, i.e. before ASF was officially detected in Estonia, made an earlier detection virtually impossible. In the study of Nurmoja *et al*.^11^, two different hypotheses were formulated. As in the present study, the authors postulated that an undetected epidemic may have occurred in the North of Estonia, which had started earlier. This may explain the different courses of the epidemics in the North and in the South. However, Nurmoja *et al*.^11^ also tested the hypothesis that the ASF strain in the North might be less virulent. Although one animal had recovered from an infection with the ASFV strain circulating in the North of Estonia, this virus still proofed to be highly virulent.

Active ASF surveillance in wild boar in Estonia started in 2012. In 2012 and 2013, according to the annual surveillance plan, it was obligatory to investigate serologically 0.5–1% of hunted wild boar, while virological investigations were not performed. In 2012, the total number of investigated wild boar in the whole of Estonia was 122; three samples were taken in area N and 21 in area S. In 2013, the total number of investigated wild boar in Estonia was 279, including six samples from area N and 65 samples from area S. Our analyses showed that even at the beginning of the epidemics in Estonia, the sample sizes in the area bordering Russia in the North were too small to have a reasonable chance of detecting ASF infections. By assuming an unknown population size and perfect specificity, it had been necessary to test at least 66 samples with a negative result to show that ASFV prevalence was below 5%. To detect the virus with a design prevalence of 1% the required sample size would have been over 300 samples (http://epitools.ausvet.com.au/content.php?page=home). When the true sample sizes mentioned above are taken into consideration, it becomes obvious, that the infection would have remained undetected, if it had been present already in 2013 or 2012. However, it must be assumed that a new emergence of ASF in a naïve wild boar population should have led to an increased mortality in wild boar. Such incidences were not reported in the years before the official outbreak in 2014. However, detecting dead wild boar might be difficult in areas with such a low population density as reported for area N^28^. In addition, the population density was even lower in the Eastern part of area N than in the other parts of the area. Accordingly, it might be practically impossible to reach the required sample sizes in areas with such a small wild boar population.

In summary, we studied the epidemiology of ASF in two areas in Estonia. The temporal and spatial differences in the course of the epidemic in the two areas suggest that the first introduction of ASF took place in the Northeast of Estonia and not, as previously assumed, in the South. This first introduction may have happened several months before Estonia was officially declared as affected by ASF.

These findings may initiate a revision and adaptation of current surveillance activities in countries that are at risk of ASF introduction, to prevent an unnoticed introduction of the disease and its spread^29^.

## Electronic supplementary material

Supplementary Material
