# Supplementary material for: Development of African swine fever epidemic among wild boar in Estonia - two different areas in the epidemiological focus
Source: Sci Rep. 2017 Oct 2;7:12562. doi: 10.1038/s41598-017-12952-w (PMC5624900; doi:10.1038/s41598-017-12952-w)
Supplement: Supplementary file 1 — Supplementary Material [file 41598_2017_12952_MOESM1_ESM.pdf]

Development of African swine fever epidemic among wild boar in Estonia - two different areas in the epidemiological focus

Imbi Nurmoja, Katja Schulz, Christoph Staubach, Carola Sauter-Louis, Klaus Depner, Franz J. Conraths, Arvo Viltrop

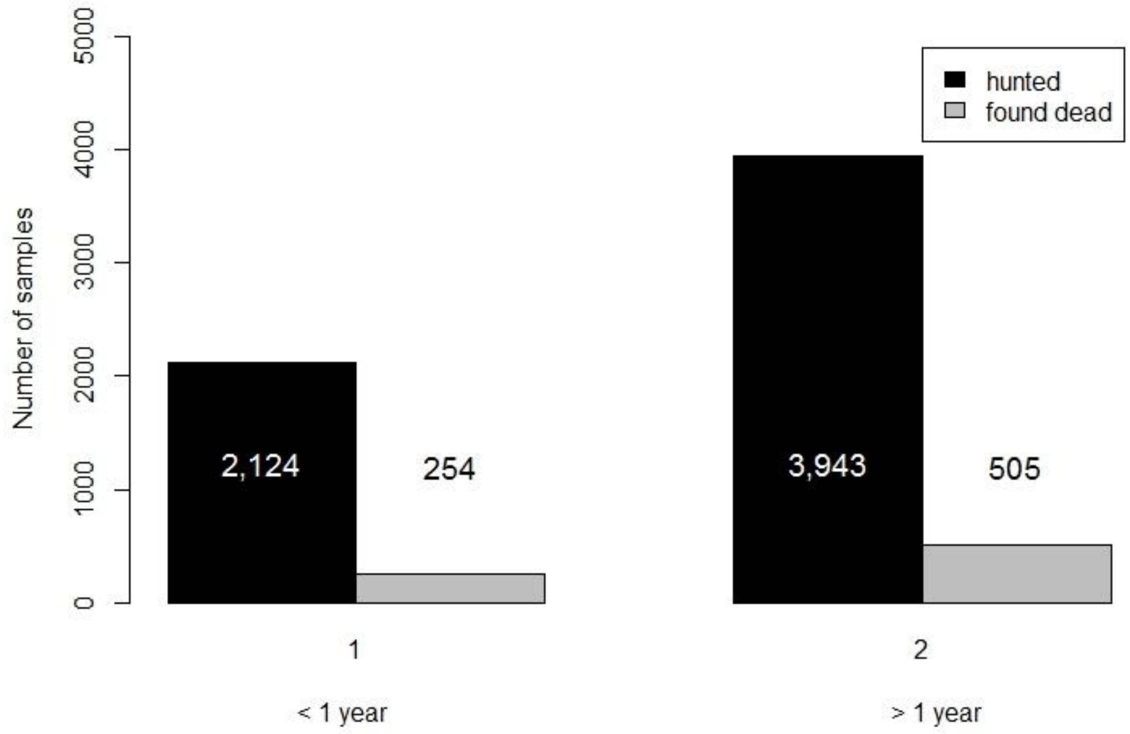

Supplementary Figure S1: Number of samples from animals hunted or found dead (carcass categories) stratified by age category (< 1 year and > 1 year).

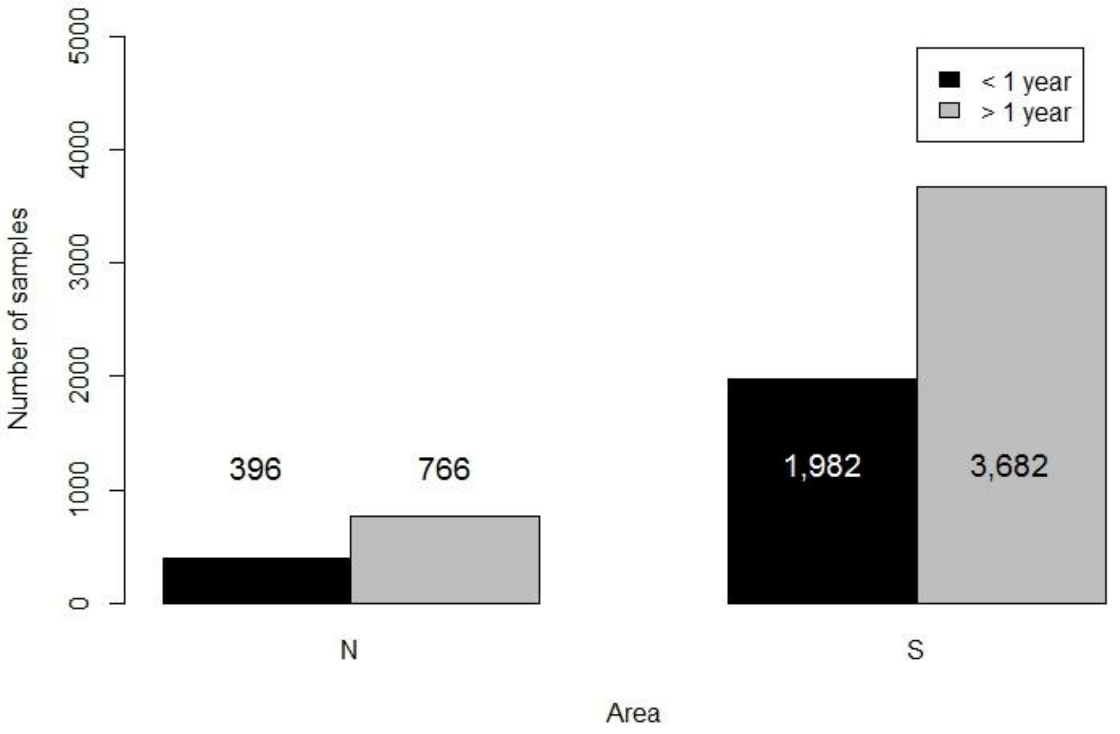

Supplementary Figure S2: Number of samples from juvenile and adult animals stratified by study area (area North [N], area South [S]).

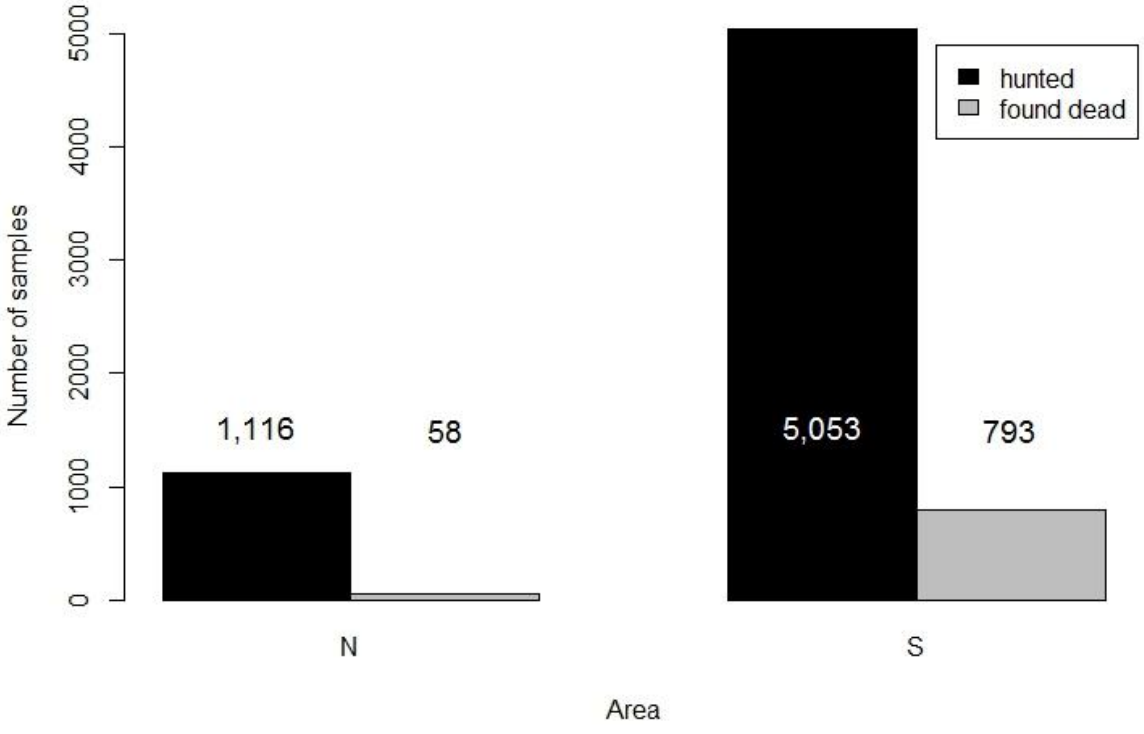

Supplementary Figure S3: Number of samples from animals hunted or found dead (carcass categories) stratified by study area (area North [N], area South [S]).

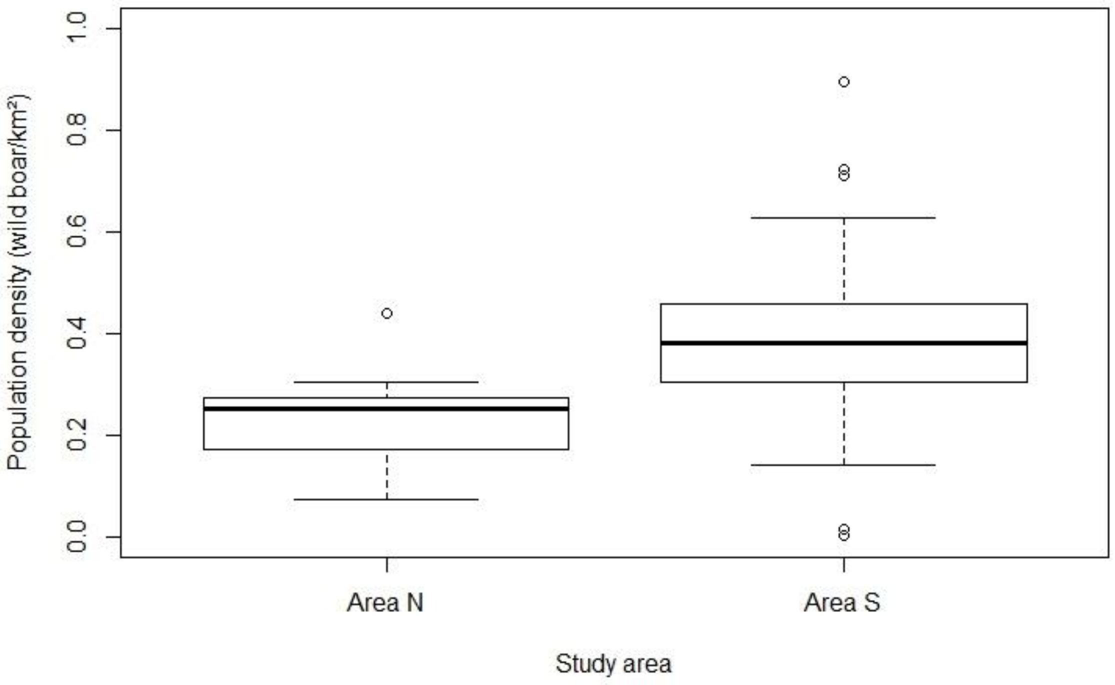

Supplementary Figure S4: Population density (number of wild boar /km²) in the study areas (area North [N], area South [S]).
